# Patterns and Drivers of Scattered Tree Loss in Agricultural Landscapes: Orchard Meadows in Germany (1968-2009)

**DOI:** 10.1371/journal.pone.0126178

**Published:** 2015-05-01

**Authors:** Tobias Plieninger, Christian Levers, Martin Mantel, Augusta Costa, Harald Schaich, Tobias Kuemmerle

**Affiliations:** 1 Department of Geosciences and Natural Resource Management, University of Copenhagen, Frederiksberg C, Denmark; 2 Geography Department, Humboldt-Universität zu Berlin, Berlin, Germany; 3 Center for Environmental and Sustainability Research (CENSE), Universidade Nova de Lisboa, Caparica, Portugal; 4 Chair for Landscape Management, University of Freiburg, Freiburg, Germany; 5 Integrative Research Institute on Transformations in Human-Environment Systems, Humboldt-Universität zu Berlin, Berlin, Germany; University of Maryland at College Park, UNITED STATES

## Abstract

Scattered trees support high levels of farmland biodiversity and ecosystem services in agricultural landscapes, but they are threatened by agricultural intensification, urbanization, and land abandonment. This study aimed to map and quantify the decline of orchard meadows (scattered fruit trees of high nature conservation value) for a region in Southwestern Germany for the 1968 2009 period and to identify the driving forces of this decline. We derived orchard meadow loss from 1968 and 2009 aerial images and used a boosted regression trees modelling framework to assess the relative importance of 18 environmental, demographic, and socio-economic variables to test five alternative hypothesis explaining orchard meadow loss. We found that orchard meadow loss occurred in flatter areas, in areas where smaller plot sizes and fragmented orchard meadows prevailed, and in areas near settlements and infrastructure. The analysis did not confirm that orchard meadow loss was higher in areas where agricultural intensification was stronger and in areas of lower implementation levels of conservation policies. Our results demonstrated that the influential drivers of orchard meadow loss were those that reduce economic profitability and increase opportunity costs for orchards, providing incentives for converting orchard meadows to other, more profitable land uses. These insights could be taken up by local- and regional-level conservation policies to identify the sites of persistent orchard meadows in agricultural landscapes that would be prioritized in conservation efforts.

## Introduction

Scattered trees are central—and dynamic—elements of many agricultural landscapes worldwide. Scattered trees have become an important object of landscape ecological research, as there is growing awareness that these ecological keystone structures govern much of the biodiversity and ecosystem services on farmlands [1–3]. In Europe, scattered trees cover as much as 55,000 km^2^ of farmlands in Portugal and Spain, 20,000 km^2^ in Greece and 3,300 km^2^ in Great Britain, but pan-European data on their spatial extent are lacking so far [4–5]. Scattered trees are threatened in many regions as the financial revenues of farmlands that integrate scattered trees are often lower than those of other, more intensive, agricultural systems [6–7]. As a result, scattered trees have mostly survived in areas only marginally suited for industrial agriculture. The remaining systems are at risk of being converted to more intensive agriculture, which typically involves the clearing of trees or the suppression of tree regeneration [1,8].

Central European orchard meadows are composed of scattered, tall fruit trees within semi-natural grasslands [9,10]. Orchard meadows are spread across 11 European countries and cover approximately 10,000 km^2^, concentrated in a belt stretching from Northern France, through Southern Germany and Switzerland to Poland [9,11–12]. Orchard meadows exhibit large habitat variation over space and time while also supporting high levels of farmland biodiversity [13–16]. They provide provisioning services, such as regionally produced drinks (juices, cider, and spirits) or forage for livestock, and are reservoirs of old landraces and cultivars [17]. Orchard meadows fulfill important cultural services—in particular recreation, scenic values and regional identity [18–19]. Moreover, they also provide critical regulating ecosystem services, such as regulating local microclimatic conditions (in particular temperature, humidity, and wind speed), reducing surface water runoff and increasing infiltration, and sequestering carbon [20–21].

Despite their unique value in terms of biodiversity and ecosystem services, orchard meadows have recently been on decline with regard to total area and number of trees [9,22–24], yet little empirical information on the loss of orchard meadows is available. Losses have been attributed to three major processes of land-use change: (1) replacement by more intensive forms of agriculture, (2) conversion into residential development areas, and (3) abandonment of orchard meadows (due to lack of profitability) and subsequent succession into shrublands or woodlands [16,22,25]. Similar declines have been reported for other scattered tree formations in Europe, such as the *montado* and *dehesa* oak woodlands on the Iberian Peninsula [26–27] and the wooded pastures of the Swiss mountains [28] and in Southeastern Europe [29].

Traditionally, land change research has focused on mapping and understanding change patterns [30], while often disregarding the causal factors of these changes [31]. Similarly, most available information on the trajectories of orchard meadows is from local case studies, essays, or qualitative descriptions, while spatially explicit studies are scarce. We are not aware of a single assessment that addressed the environmental, economic, and social drivers of orchard meadow change in a quantitative fashion. This is unfortunate because such knowledge is urgently needed to develop effective policies to halt orchard meadow loss and to safeguard their multiple ecosystem services.

To fill these research gaps, our overall aims were (1) to map and quantify the decline of orchard meadows for the 1968-2009 period for a region in Southwestern Germany and (2) to identify the underlying environmental, demographic, and socio-economic drivers of this decline. We hypothesized that the decline of orchard meadows should be higher:

- in areas of smoother slopes and richer soils (H1),
- in areas where agricultural intensification was stronger (H2),
- in areas where smaller plot sizes and fragmented orchard meadows prevail (H3),
- in areas of increasing population density, near settlements, and near infrastructure areas (H4), and
- in areas of lower implementation levels of conservation policies (H5).

## Materials and Methods

### Study Area

**Fig 1:**
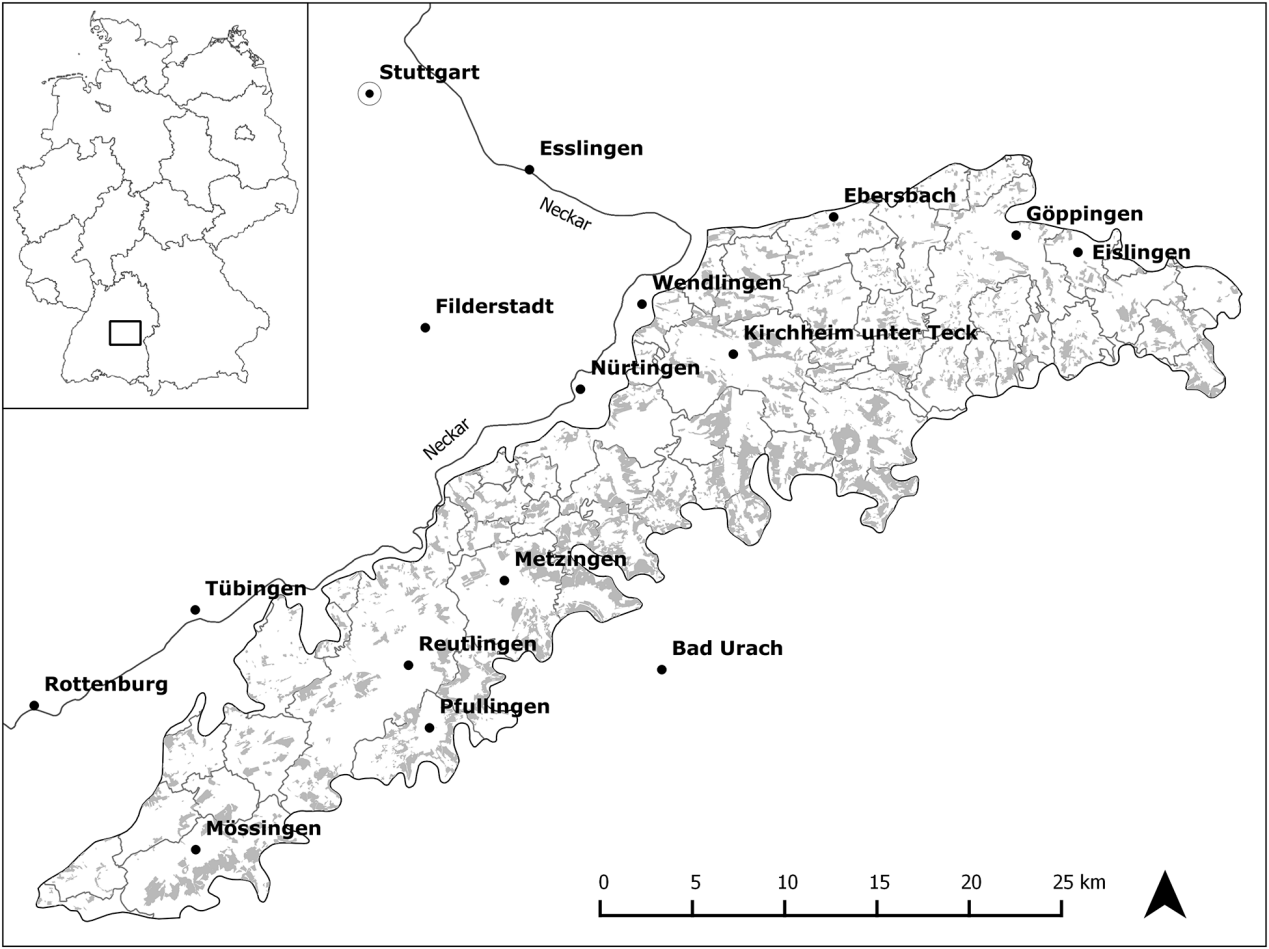
Map of the study area within Germany. Grey areas indicate orchard meadow land cover in 2009.

Our study targeted orchard meadows within a landscape of 70,109 hectares (ha) in the foothills of the Swabian Alb mountain range, in the state of Baden-Württemberg, Southwest Germany (48.23–48.44°N and 8.55–9.45°E, Fig 1). The study area is divided into 71 municipalities and five counties and is composed of agricultural land (60.5%), forest (20.3%), and built-up land (19.2%). This area has experienced important socio-economic changes since 1968, especially with decreases in number of farms and increases in population (Table 1). Our study included a total of 9,170ha of orchard meadows. Predominant tree species are diverse varieties of apple (*Malus domestica*), plum (*Prunus institia*, *Prunus domestica*), cherry (*Prunus avium*), pear (*Pyrus comunis*), and walnut (*Juglans regia*). Orchard meadows characteristically occur in belts around villages and range from the valley bottoms to the lower and middle elevations of the surrounding hills. They are predominantly under private, small-scale ownership (mean parcel size: 0.22ha ± 0.01ha S.E.), but in some places they are owned by local municipalities. Large segments of the area’s orchard meadows are included under different protected-area categories: Nature reserves cover 3.3% of the orchard meadow area and offer a high degree of protection, exhibiting location-specific conservation management and restrictions on agricultural and forestry uses. Protected landscapes prevail on 42.4% of the area, where regulations are more focused on landscape scenery usually interdicting conversion of agricultural land into built-up land. The climate is temperate, with warm summers and cool winters. Average annual temperature is 9.5°C (low of 0.7°C in January and high of 18.8°C in July; Metzingen Weather Station); average annual precipitation is 867 mm (Nürtingen-Reudern Weather Station) [32]. The area comprises the foothills of the Swabian Alb mountain range and is characterized by an ascending slope over lower and middle Jurassic formations. Loess loam is the prevailing sediment, providing fertile soils for agriculture [33].

**Table 1:** Key socio-economic variables and their changes in the study area.

Variable	1968/79 (t_1_)	2009 (t_2_)	Change (t_2/_ t_1_)
Full-time farms (t_1_: 1979)	1565	398	25.4%
Part-time farms (t_1_: 1979)	2956	725	24.5%
Horticultural farms (t_1_: 1979)	259	81	31.3%
Livestock farms (t_1_: 1979)	3690	959	26.0%
Population (t_1_: 1968)	656,306	801,223	122.1%
Residential buildings (t_1_: 1968)	105,916	177,813	167.9%

Reference years t_1_ vary due to differences in the availability of statistical data (source: Baden-Württemberg Statistical Office).

### Data

We assessed changes in orchard meadow cover between 1968 and 2009 through evaluation of aerial photographs (1968) and digital orthoimages (2009) that were provided by the State Agency for Spatial Information and Rural Development of Baden-Württemberg. The selection of the two reference years was based on data availability. The 1968 images were taken from the first state-wide survey flight and comprised a set of 126 images (grayscale, 23 x 23 cm, scale: 1:12,000) that were scanned in 1,270 dpi. The images were georeferenced to the Gauss-Krüger grid system (datum: D_Deutsches_Hauptdreiecksnetz, zone: 3), using 15–30 ground control points for each image. Root mean squared errors were below 7 m for all images. The ground resolution of the 1,098 digital orthoimages (RGB, Gauss-Krüger projection) was 25 cm. Digital 1:25,000 topographic maps from the State Agency for Spatial Information and Rural Development of Baden-Württemberg were used for determining broad land cover units (reference year: 2009).

### Mapping Loss and Persistence of Orchard Meadows

For the analysis of orchard meadow loss and persistence, we derived orchard meadow maps for 1968 and 2009 from the aerial images, defining orchard meadows as all grassland plots on which trees were scattered with densities of 20 to 100 trees per ha. As we were mainly interested in the changes of extensive orchard meadows and less so in isolated fruit trees, our minimum mapping unit was 0.1ha. We mapped 1,548 orchard meadow polygons for the 1968 layer and 1,458 for the 2009 layer, making use of the distinct texture and tone in the aerial images that clearly separates orchard meadows from other land uses [34]. Overlaying the polygon maps for 1968 and 2009, we categorized ‘lost orchard meadows’ as those locations covered by orchard meadows in 1968 but not in 2009. Meanwhile, ‘persistent orchard meadows’ were orchard meadows that existed in both 1968 and 2009. The annual rate of orchard meadow loss was calculated as follows [35]:

$$r=\left(\frac{1}{2009-1968}\right)*\text{ln}\left(\frac{Area\;\text{2009}}{Area\;\text{1968}}\right)$$ (1)

Data were converted into raster format and resampled to a spatial resolution of 25 m. We randomly selected 1,997 points, which formed the sample for the statistical analyses: 435 points for ‘lost orchard meadows’ and 1,562 points for ‘persistent orchard meadows’ (S1 Table).

### Explanatory Variables and Their Hypothesized Influence

We identified potential explanatory variables based on expert interviews and regional literature [16,22,36–37]. In accordance to our five hypotheses, we grouped our explanatory variables into: biophysical factors, trends in agricultural structure, land ownership structure, trends in population and settlement, and conservation measures (Table 2).

**Table 2:** Explanatory variables in five groups that were included in the analysis.

Variable	Spatial scale	Unit	Time period	Expected influence	Variable type	Source
*Biophysical factors*						
Slope	Pixel	°	-	+	Continuous	RIPS
Soil quality	Pixel	1–5	-	–	Ordinal	RIPS
*Trends in agricultural structure*						
Change in number of full-time farms	Municipality	%	1979–2010	–	Continuous	SL-BW
Change in number of part-time farms	Municipality	%	1979–2010	+	Continuous	SL-BW
Change in number of horticultural farms	Municipality	%	1979–2010	+	Continuous	SL-BW
Change in number of livestock farms	Municipality	%	1979–2010	+	Continuous	SL-BW
*Land ownership structure*						
Parcel size	Pixel	ha	2009	+	Continuous	ALK
Size of orchard meadow plot	Plot	ha	1968	+	Continuous	Own calculations
Perimeter-area ratio of orchard meadow plot	Plot	m m^-2^	1968	–	Continuous	Own calculations
*Trends in population and settlement*						
Population change	Municipality	%	1968–2009	–	Continuous	SL-BW
Change in number of residential buildings	Municipality	%	1968–2009	–	Continuous	SL-BW
Share of people ≥ 65 years among population	Municipality	%	1970	–	Continuous	SL-BW
Change in settlement and traffic area	Municipality	%	1980–2009	–	Continuous	SL-BW
Distance to urban settlements	Pixel	km	2009	+	Continuous	Own calculations
Distance to main roads	Pixel	km	2009	+	Continuous	Own calculations
Distance to secondary roads	Pixel	km	2009	+	Continuous	Own calculations
*Conservation measures*						
Situated within nature reserve	Pixel	yes / no	2010	+	Nominal	LUBW
Situated within protected landscape	Pixel	yes / no	1969	+	Nominal	LUBW

Expected influence refers to the hypotheses specified in the introduction: + means that a higher value for a variable would explain persistence of orchard meadows;—means that a higher value for a variable would explain orchard meadow loss.

RIPS = Spatial Information and Planning System of Baden-Württemberg, https://www.lubw.baden-wuerttemberg.de/servlet/is/16129/.

SL-BW = Baden-Württemberg Statistical Office, http://www.statistik.baden-wuerttemberg.de/.

Automated Land Registration Maps, https://www.lgl-bw.de/lgl-internet/opencms/de/05_Geoinformation/Liegenschaftskataster/

LUBW = State Institute for Environmental Protection, http://www.lubw.baden-wuerttemberg.de/.

We included two biophysical factors to account for conditions of soil and topography. We calculated slope using a digital elevation model with a resolution of 5 m from the Spatial Information and Planning System of Baden-Württemberg. Soil quality describes the ecological productivity potential of agricultural land as a function of local soil properties, topography, and climate, expressed along an ordinal scale from 1 to 5.

The second group of variables is related to agricultural structure at municipal scale. We derived these variables—changes in the numbers of full-time, part-time, horticultural, and livestock farms between 1979 and 2010—from the regional statistics database of the Baden-Württemberg Statistical Office.

Third, land ownership structure, and in particular land consolidation, exerts potential influence on orchard meadow dynamics [22,36]. To measure land property regimes, we derived parcel sizes from the Automated Land Registration Maps (ALK) of Baden-Württemberg. We also calculated the size of each orchard meadow plot, as we expected that small and more-complex patches, as expressed by plot size and perimeter-area ratios of orchard meadows, would be more likely to be converted than larger and less-complex ones.

In the fourth group, we assembled variables on population and settlement patterns at the municipal level, for which the most detailed demographic statistics were available from the Baden-Württemberg Statistical Office from the year 1968 onwards. We included population change, change in number of residential buildings, percent of population ≥ 65 years, and change in settlement and traffic area. Additionally, we calculated distance values from each orchard meadow plot to urban areas and major and secondary roads.

In the fifth group, we used data on conservation measures. We expected that orchards without formal protection would have higher conversion risk than those within nature reserves or protected landscapes. Maps of nature reserves and protected landscapes (reference years: 2010 and 1969) were obtained from the State Institute for Environmental Protection (LUBW).

For our initial exploration of the data, we performed univariate Spearman rank correlation analysis to determine whether orchard meadow loss was spatially associated with each of the 15 explanatory variables assessed. For this analysis, we calculated the percentage of orchard meadow loss in each of the study area’s 64 municipalities that included orchard meadows.

### Boosted Regression Trees

The main part of our analysis was based on boosted regression trees (BRTs), a machine-learning technique that combines the concept of decision trees with that of gradient boosting [38–41]. From our perspective, BRTs offer several advantages, as they (1) can use any type of input data and are not dependent on normally distributed explanatory variables; (2) can handle non-linear relationships and are not sensitive to outliers, missing data, and collinearity between explanatory variables; (3) tend to be robust towards overfitting due to the random exclusion of a certain fraction of input data (bagging); and (4) generally obtain higher model fits and predictive accuracies compared to conventional statistical approaches [38–41]. Furthermore, BRTs enable the assessment of interactions between predictors and efficient selection of relevant variables [38], alleviating the problem of bias in variable selection and relative importance for recursive partitioning methods [42] by employing random observation selection contrasting with the random feature selection of random forests [43]. Due to these factors, BRTs are a highly useful analytic tool to explore complex relationships, such as those between orchard meadow loss and its manifold social-ecological drivers.

We performed our analysis using the *dismo* package [44] in R [45]. For the calibration of BRT models, it is necessary to specify four main parameters: bag fraction, tree complexity (tc), learning rate (lr), and number of trees. The bag fraction determines the share of input data randomly withheld while fitting each new decision tree. Following Friedman [40], we chose a bag fraction of 50%. Tree complexity defines the maximum number of splits in each decision tree and, thus, determines the levels of interaction between the explanatory variables. The learning rate scales down the contribution of each additional tree to the entire BRT model. Both tree complexity and learning rate strongly influence model performance. It is thus important to find the parameter combination with the minimum predictive error. To do so, we performed a systematic sensitivity analysis, testing all combinations of tree complexity values form 1 to 9 and learning rates from 0.1 to 0.001. To compensate for stochastic bias, we calculated the row and column averages of tenfold cross validated ROC (receiver operating characteristics) scores and Pearson correlation coefficients and chose the optimal parameter combination for this analysis (i.e., tree complexity = 8; learning rate = 0.01). The number of trees defines the number of single decision trees included in the model and is a measure for the model complexity. We used the *gbm*.*step* routine of the *dismo* package to determine the appropriate number of trees. To obtain measures of model performance, we calculated correlation coefficients (between observed and modeled values) and ROC scores for both training and testing data as well as the percentage of deviance explained by the model. Based on predicted and observed values for orchard meadow loss, we computed the true positive rate, indicating correctly classified loss locations, the true negative rate, indicating correctly classified non-loss locations, as well as model accuracy and precision.

### Model Interpretation and Data Analysis

Relative importance [40] is a measure of the influence of each explanatory variable on a model’s outcome, independent from the model’s performance itself (i.e., ROC score or percent deviance explained). Relative importance is estimated by counting the number of times a variable is selected in a tree, weighted by the squared improvements, and averaged over all trees. Relative importance across all variables is then standardized to sum up to 100%. Variables with a relative importance smaller than expected by chance (for 18 variables as in our case: 1/18 or 5.56%) were not interpreted further [46]. To visualize the influence of each explanatory variable on the probability of orchard meadow loss, we used partial dependency plots (PDPs) which depict the influence of an explanatory variable while keeping all other variables constant.

## Results

### Patterns and Rates of Orchard Meadow Loss

**Fig 2:**
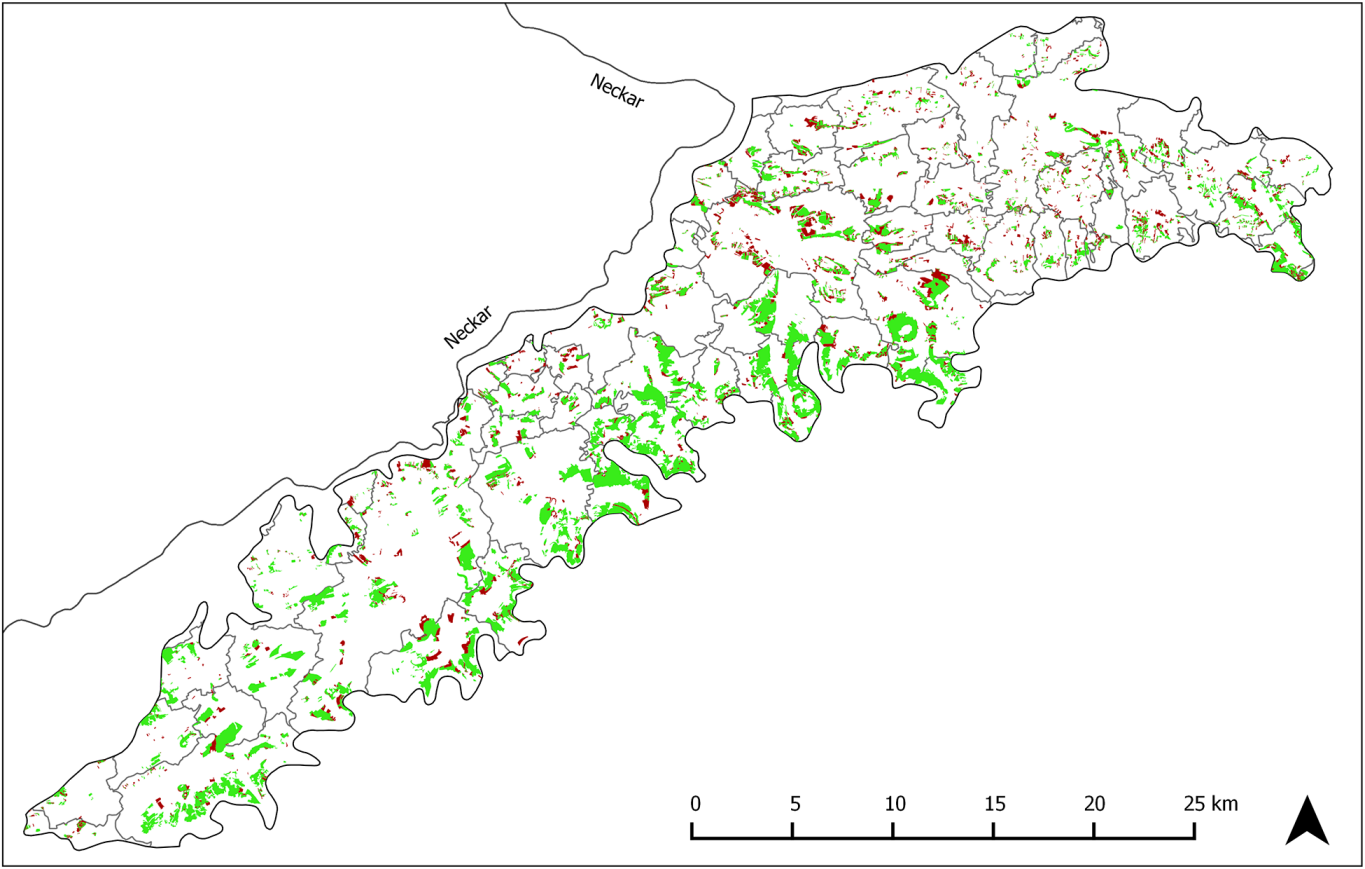
Orchard meadow change map, 1968–2009. Orchard meadows in green have remained persistent, while those in red have been lost.

**Fig 3:**
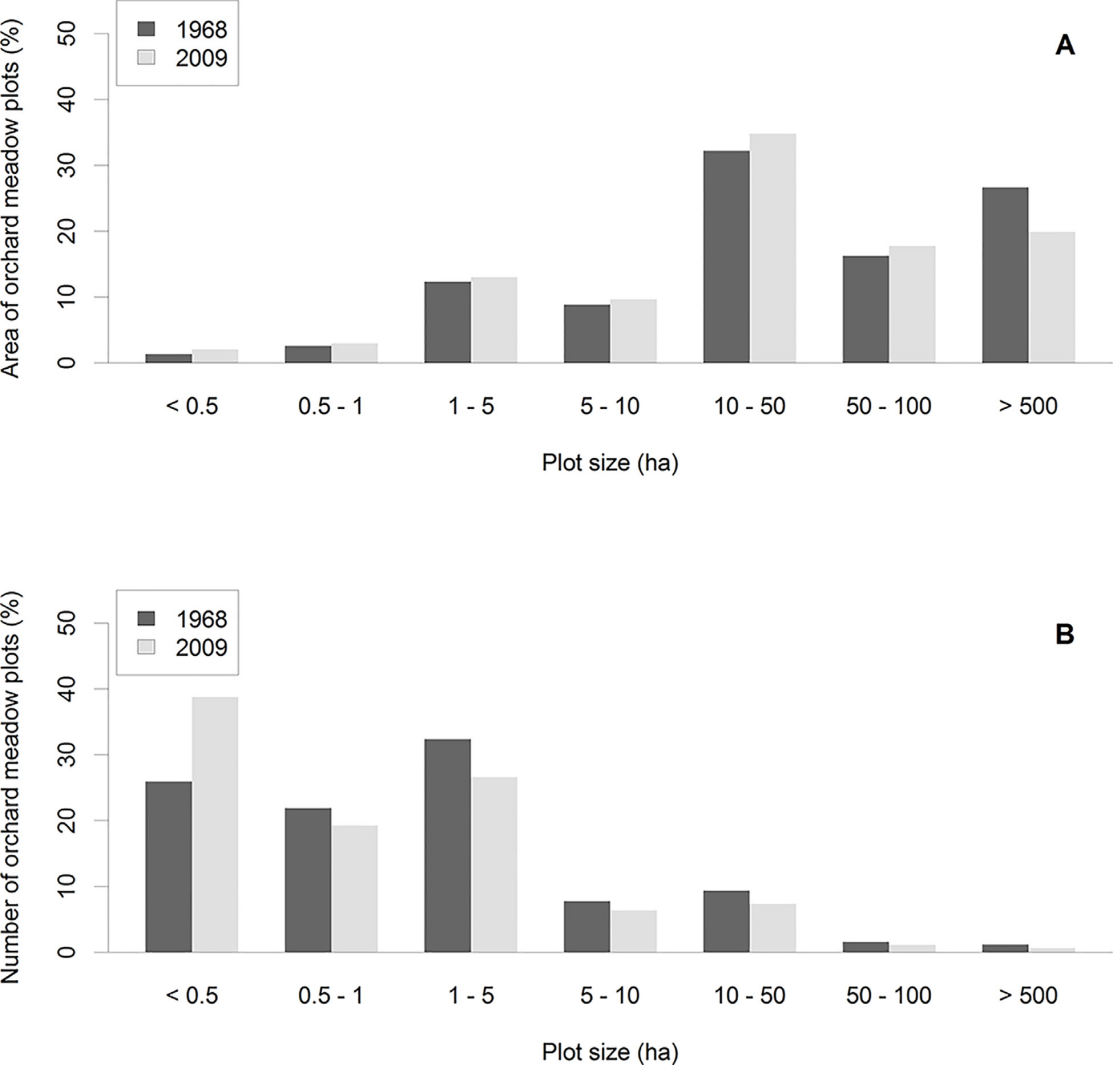
Percentage of total area (above) and number (below) of orchard meadow plots, by plot size in ha.

The amount and spatial distribution of orchard meadow loss (i.e. areas where orchard meadows existed in 1968 but not in 2009) were substantial (Fig 2). In 1968, 15.6% of the study area was covered by orchard meadows, but cover had decreased to 12.1% by 2009. The annual rate of orchard meadow loss was 0.62%. The average size of an orchard meadow plot was 6.17ha in 1968 and 4.61ha in 2009. More than 84.6% of the orchard meadow plots were smaller than 5ha in 2009; however, 82.0% of the orchard meadow cover was in plots of 5ha area or larger (Fig 3).

Although most orchard meadows persisted (7,090.6ha / 78%) throughout our study period, a considerable share (2,079.4ha / 22%) was converted to other land uses. These losses were not distributed uniformly across the landscape: Within the 64 municipalities included in our study, 12 municipalities had orchard meadow area losses below 10%, 25 municipalities exhibited losses of between 10% and 30%, while 27 municipalities experienced losses of more than 30%. By 2009, 382 orchard meadow plots (of 1,548 plots in 1968) were completely removed, whereas 820 plots were downsized by more than 100m^2^. Most of the area loss occurred due to conversion to tree-less agriculture (1,301.7ha / 63%) and to built-up land (672.9ha / 32%). Conversion to forest / shrubland or other land use classes played only a minor role (90.6ha / 4% and 14.2ha / 1%, respectively). Similarly, 67.2% of the lost orchard meadow plots were converted to tree-less agricultural land, 24.5% to built-up land, and only 13.8% to forest or other land use classes.

### Determinants of Orchard Meadow Loss

**Fig 4:**
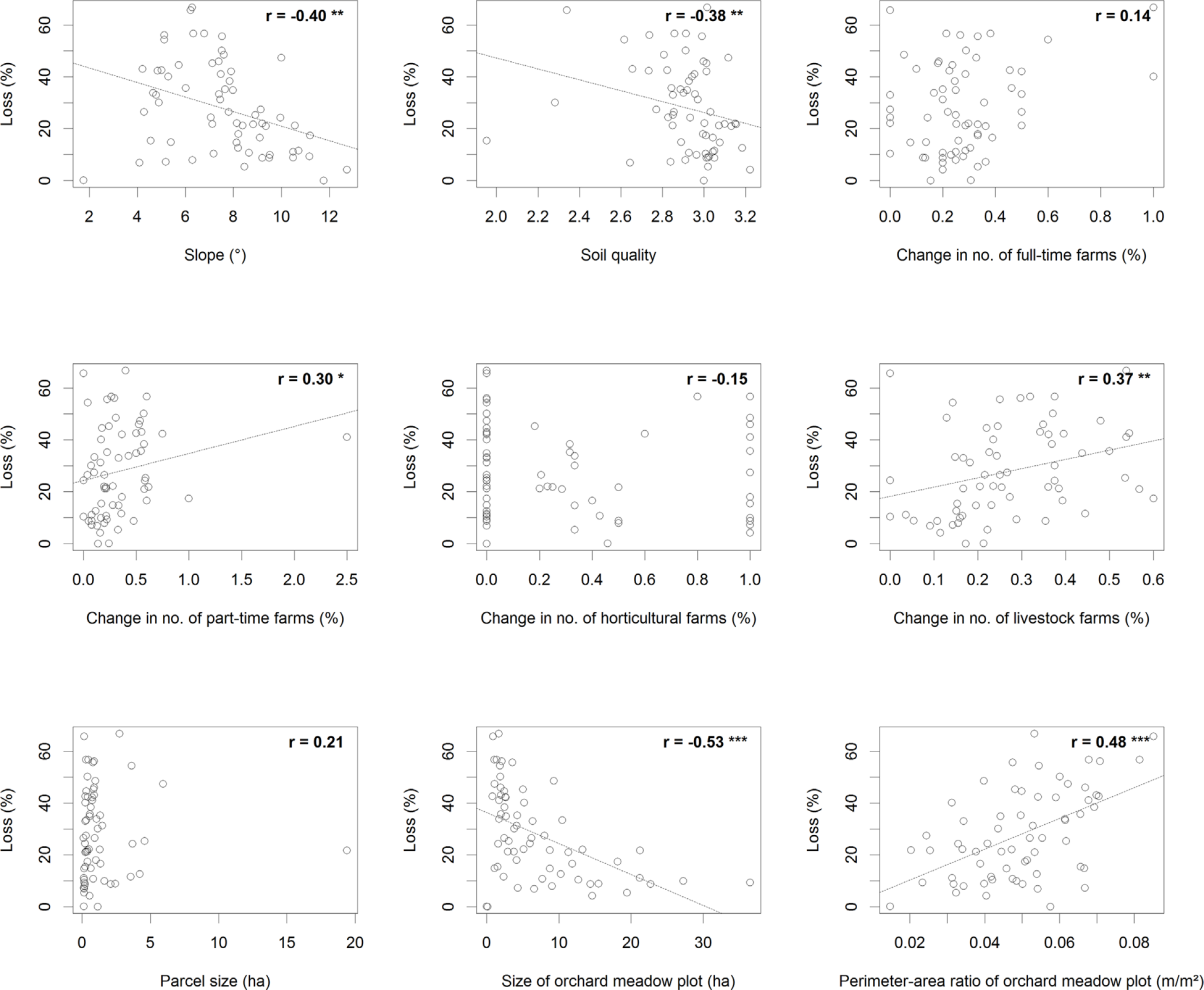
Relationships between orchard meadow loss (%) and continuous explanatory variables for three groups: biophysical variables, trends in agricultural structure, land ownership structure (n = 64 municipalities). Numbers specify Spearman rank correlation coefficient r. * indicates a significance level of p<0.05, ** p<0.01, and *** p<0.001.

**Fig 5:**
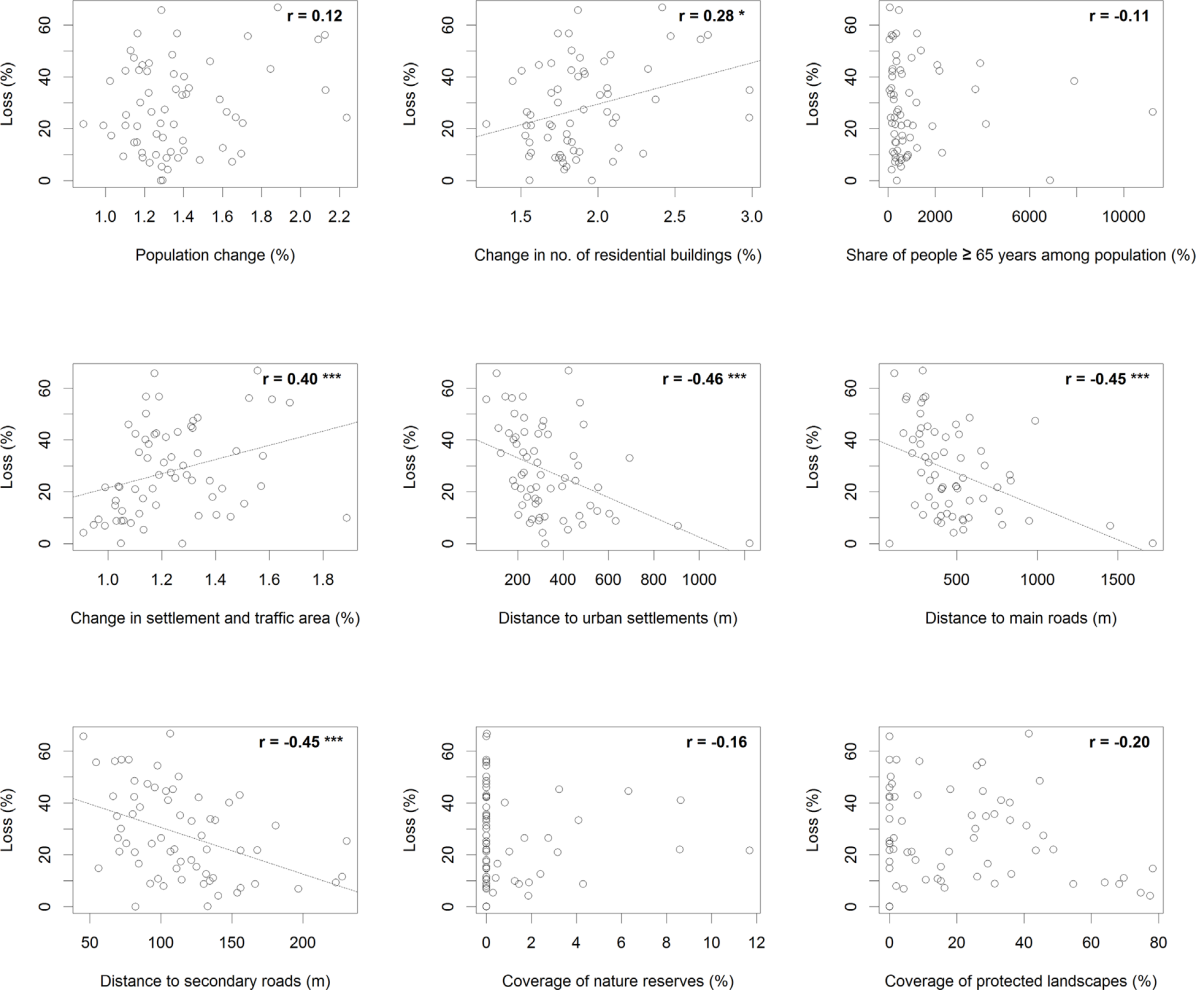
Relationships between orchard meadow loss (%) and continuous explanatory variables for the trends in population and settlement and conservation measures groups (n = 64 municipalities). Numbers specify Spearman rank correlation coefficient r. * indicates a significance level of p<0.05, ** p<0.01, and *** p<0.001.

Univariate correlation analyses between loss rates and our continuous explanatory variables revealed highly significant (p<0.001) positive relationships for perimeter-area ratio (r = 0.48) and change in settlement and traffic area (r = 0.40) (Figs 4 and 5). Highly significant negative relationships were found for size of orchard meadow plot (r = -0.53), distance to urban settlements (r = -0.46), distance to main roads (r = -0.45), and distance to secondary roads (r = -0.45) (Figs 4 and 5).

Our BRT model explained 29.3% of the total variation in the probability of orchard meadow loss (Table 3). Even though the explanatory power of the model was moderate, the cross-validated ROC score (0.842) indicated that our model was well-suited to predict orchard meadow loss (see S2 Table for a confusion matrix of predicted and observed values for orchard meadow loss). The accuracy and precision of the model were high, at 78% and 91%, respectively. The hit-ratio for predicting loss (true positive rate) was moderately high (63%), while prediction accuracy of non-loss locations was very high (98%, Table 4 and S2 Table).

**Table 3:** Training and validation performance of the model.

Measure	Value
Number of trees	720
cv r	0.57
cv r^2^	0.33
cv ROC	0.842
Mean total deviance	1.048
Mean residual deviance	0.647
Estimated cv deviance	0.759
% deviance explained	29.3

**Table 4:** Specification of accuracy, true and false positive rate, true and false negative rate, and precision.

Measure	Formula	Value
Accuracy (ACgmean)	ACgmean = ROOT(TPR*TNR)	0.78
True positive rate (TPR)	TPR = TP/(FN+TP)	0.63
False positive rate (FPR)	FPR = FP/(TN+FP)	0.02
True negative rate (TNR)	TNR = TN/(TN+FP)	0.98
False negative rate (FNR)	FNR = FN/(FN+TP)	0.37
Precision (P)	P = TP/(FP+TP)	0.91

Variables related to distance to settlements, secondary, and major roads, as well as those related to geometric characteristics of orchard plots (plot size, parcel size, plot perimeter-area ratio) plus slope had a combined relative importance of more than 75% (Table 5). Distance to settlements was the most important explanatory variable in predicting orchard meadow loss with a relative importance of 26.2%. Other variables were less important than expected by chance (5.56%). Identified variable interactions were relatively homogenous and generally not very strong (S3 Table).

**Table 5:** Relative importance values of explanatory variables.

Rank	Explanatory variable	Relative importance
1	Distance to urban settlements	26.2%
2	Parcel size	11.3%
3	Size of orchard meadow plot	10.4%
4	Perimeter-area ratio of orchard meadow plot	8.4%
5	Slope	6.8%
6	Distance to secondary roads	6.5%
7	Distance to major roads	6.1%
8	Change in number of livestock farms	4.5%
9	Change in number of full-time farms	3.0%
10	Change in settlement and traffic area	2.9%
11	Population change	2.8%
12	Change in number of residential buildings	2.7%
13	Change in number of part-time farms	2.6%
14	Share of people ≥ 65 years among population	2.4%
15	Soil quality	2.1%
16	Change in number of horticultural farms	1.3%
17	Situated in nature reserve	0.1%
18	Situated in protected landscape	0.0%

**Fig 6:**
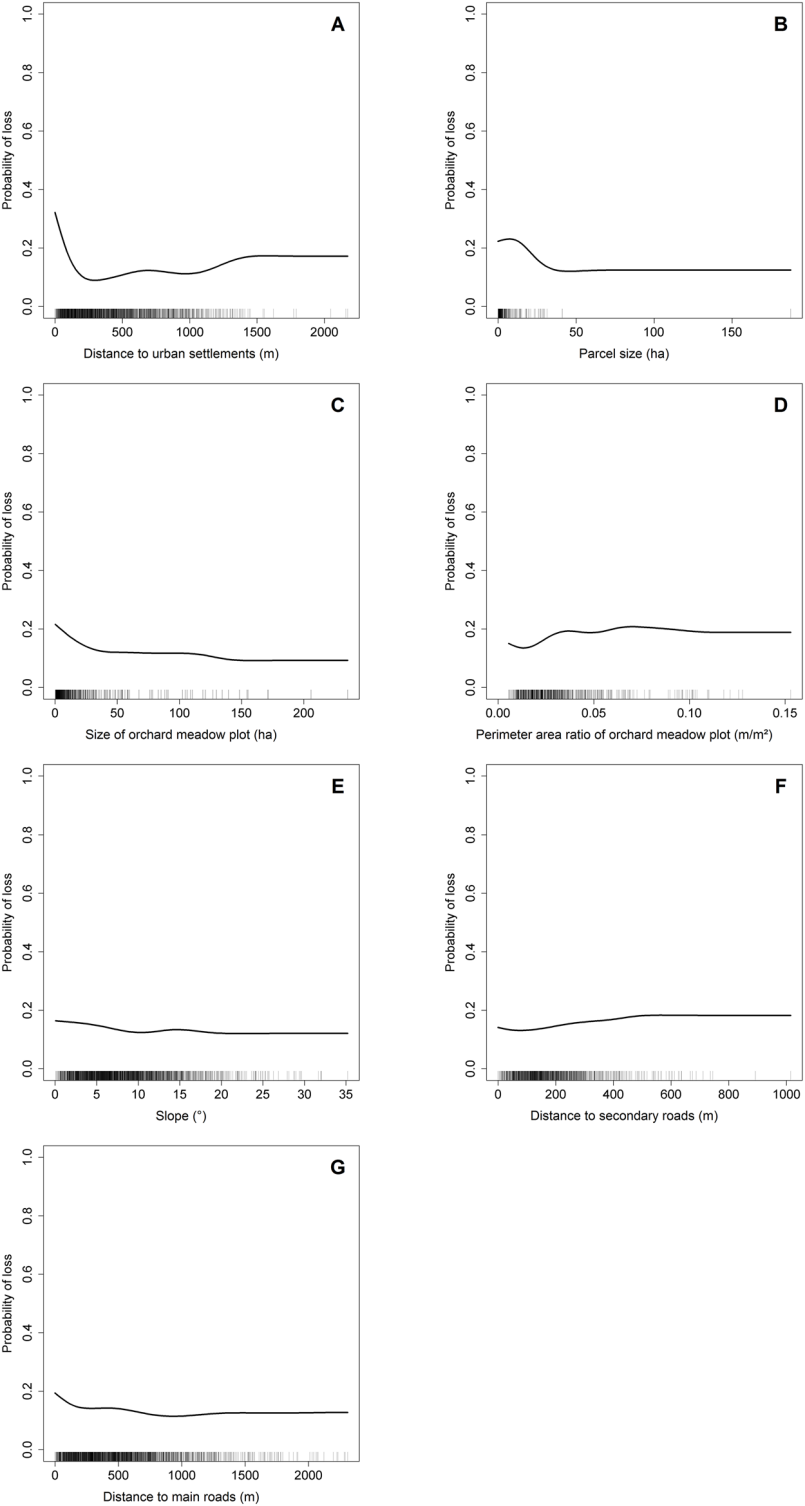
Partial dependency plots (PDPs) for the seven most important variables of the study.

The partial dependency plots (Fig 6) of the seven explanatory variables with relative importance values above the 5.56% threshold provided further insights in the type of relationship between each explanatory variable and meadow orchard loss. The probability of orchard meadow loss showed a significant decrease within the first 300m around settlements and a slight increase for areas located further than 1,000m away (Fig 6A). Increasing parcel size (Fig 6B) generally decreased the chance of loss, and plot size (Fig 6C) had a similarly negative impact. Especially for plots smaller than 40ha, the chance of loss increased significantly with decreasing size. The probability of loss generally increased with perimeter-area ratio (Fig 6D). This was especially the case for perimeter-area ratios between 0.02 and 0.04. The probability of loss decreased slightly with increasing slope (Fig 6E). Meanwhile, the distance measures related to infrastructure showed a converse behavior: The probability of loss is higher close to main roads (Fig 6G), whereas it has a nonlinear curve regarding the distance from secondary roads (Fig 6F).

## Discussion

Orchard meadows provide a wide range of ecosystem services and are of great importance for farmland biodiversity conservation, yet have been declining throughout Europe recently [23]. Understanding the rates, spatial patterns, and determinants of these declines is key for developing effective policies to halt orchard meadow losses. We combined a landscape-scale assessment of orchard meadow change in Southern Germany with a spatially explicit analysis of the driving forces of change. We found that, over a 41-year period, about 22% of orchard meadow area and 25% of orchard meadow plots have been lost. The rate of orchard meadow loss that we measured (0.62% per year) was smaller than previous estimates for other regions: Loss of scattered fruit trees in Switzerland, for example, amounted to 70% from 1951 to 1991 (3.01% per year) [11]. Between 1968 and 1988, orchard meadows declined by 50% (3.47% per year) in Austria [47]. In an Eastern German landscape, mean annual loss rates from 1964 to 2008 were 1.05% [34], whereas in France orchard meadows diminished by 2.80% per year (from 259,000 ha to 144,000 ha) between 1982 and 2003 [48]. Meanwhile, it was estimated that, in the state of Baden-Württemberg (Southern Germany), the number of scattered fruit trees decreased from 18.0 million to 9.3 million between 1965 and 2005 (1.65% per year) [25]. The lower rate of orchard meadow loss found in our study may be explained by the fact that our starting point of 1968 was not when orchard meadow conversion began. Rather, scattered fruit trees had already been removed from the study area during several intensive clearing campaigns in the 1950s [16]. Also, loss rates of individual scattered trees are generally higher than those of orchard meadow area [49].

The direct causes of orchard meadow loss were mainly, as can be expected in a densely populated and intensively cultivated landscape, related to agriculture and urbanization, while land abandonment was less important. Exploratory analysis showed significant (p<0.05) correlations with orchard meadow loss for 11 of the 18 explanatory variables (Figs 4 and 5). The BRT method allowed us to identify the most influential variables used for predicting orchard meadow loss from our given set of potentially important explanatory variables: distance to urban settlements, parcel size, size of orchard meadow plot, perimeter-area ratio of orchard meadow plot, slope, distance to secondary roads, and distance to major roads (in decreasing order of variable importance). The other indicators showed much less impact (Table 5).

We set out to test five hypotheses regarding the influence of different potential drivers of orchard meadow loss. Our first hypothesis (H1) was that orchard meadow loss would be higher in areas of smoother slopes and richer soils, as these factors determine the suitability of land for conversion to more profitable uses [10,16]. Indeed, the BRT method identified slope as influential driver of orchard meadow loss, but soil conditions were less important. Loss rates were higher in flatter areas, probably because such areas are more prone to agricultural intensification. Soil quality is often a relevant land-use driver at the local scale, especially in Mediterranean climates [50–51]. Here, our BRT analysis did not find any important influence of soil quality on orchard meadow loss at the regional scale—possibly because soil quality does not vary strongly in the region [33].

According to hypothesis H2, we expected higher orchard meadow losses in areas where agricultural intensification was stronger, as expressed in agricultural structure trends [28]. Indeed, changes in the number of livestock farms and—to a lesser degree—changes in the number of part-time farms were significantly related to orchard meadow loss in the correlation analyses (Fig 4). However, most of these relationships were weak, and the BRT approach did not identify factors related to agriculture as influential drivers of orchard meadow loss (Fig 6). This is probably a consequence of the lack of plot-level information on farming systems and orchard meadow management. Also, orchard meadows are frequently managed by small-scale, hobby farmers, who are not accounted for in official agricultural statistics [9].

We had further hypothesized that larger orchard meadow losses would appear in areas where smaller land parcel and orchard meadow plot sizes prevail and where orchard meadows are more fragmented, as expressed by higher perimeter-area ratios (H3). The BRT analysis demonstrated that orchard meadow losses were indeed higher for smaller land parcels (<40 ha), which may be related to the issues of lower profitability and more difficult management of these parcels. Additionally, areas of small-scale and fragmented land ownership have frequently undergone land-consolidation efforts [22,36]. However, the direct role of parcel size and the indirect role of land-consolidation campaigns in the loss (or improved management) of orchard meadows deserve more attention. Orchard meadow plots that were smaller and more fragmented were also more likely to be converted, again possibly because they had become less profitable and their management incurred larger opportunity costs than that of larger plots. In larger orchard meadow plots, profitability may have been high enough to prevent conversion to other land uses.

According to H4, orchard meadow loss should be linked to increased population density and proximity to settlements and infrastructure. We expected this as many orchard meadows are situated in green belts around settlements, and many villages and small towns in our study area experienced strong population and settlement-area growth over the past 40 years. The most important finding from our BRT model was that loss rates were highest in direct proximity to settlements, emphasizing the role of housing development on the removal of orchard meadows. Our BRT model did not link orchard meadow loss to population and settlement trends. This was probably due to effects of scale, as trends in population and settlement were only available at municipality level. However, the distance of an orchard meadow to both major and secondary roads was important in describing orchard meadow loss. Loss rates were higher close to main roads, as many fruit trees have been cleared in consequence of road construction activities.

Our H5 predicted higher orchard meadow losses in areas of lower levels of conservation policy implementation. However, our study did not find an influence from conservation measures (specifically, official designation of nature reserves and protected landscapes) on the rate of orchard meadow loss. Nature reserves are small in number and coverage in the study area, and protected landscapes offer only weak levels of protection. Also, institutional analysis has highlighted how designations of nature reserves or protected landscapes often fail to safeguard continued management interventions (such as pruning and extensive mowing), which are essential for the conservation of orchard trees [52]. We think this may be the main reason why we found no difference in loss rates between protected and unprotected land. Financial incentive programs may, therefore, be an important complement to protected area designations, but existing agri-environmental measures have often been insufficient for making orchard meadows profitable [15].

As we have carried out the first spatially explicit, quantitative assessment of the driving forces behind orchard meadow loss in Europe, a few sources of uncertainty need to be discussed. In terms of representativeness, our study covered an orchard meadow area that corresponds to approximately 10% of the state-wide orchard meadow surface [25], and we focused on the largest contiguous orchard meadow area in the state. As our analysis has shown that smaller and more fragmented orchards are more likely to be lost, overall loss rates in other areas of the state may be higher. The approach to classify tree cover visually on aerial photographs and orthoimages was a simple and effective way to analyze orchard meadow dynamics, as there generally is a strong contrast between scattered fruit trees and other land covers (cf. [28]). However, this approach only informs on changes in the extent of orchard meadows, but not about changes in stand densities, stand ages, management intensity, or other features within orchard meadows, of conservation importance [13,53–54].

The explanatory power of our model was moderate, similar to other studies of meadow loss [55–56], leaving a major part of variability in loss probabilities unexplained, which needs to be considered when interpreting the relative importance of predictors. Two reasons may be behind this: (1) Our biophysical, land-ownership structure, and conservation indicators were acquired at pixel or plot levels. However, some of the population and settlement indicators and all indicators related to agricultural structure were only available at the municipal level, which may have diluted the explanatory power of our model; (2) There may be important drivers that we could not include due to limitations regarding available indicators. In particular, management intensity and farming styles—defined by land-user attitudes, farming objectives, and economic success—may play a critical role [57]. Hence, relative variable importance is strongly linked to the set of explanatory variables used in our model and, consequently, neglects the possible importance of variables that were not included.

In addition to that, the explanatory power of our BRT model was lower for the orchard meadow loss class than for the stable orchard meadow one. This is likely due to two factors. First, area proportions differed vastly, with the stable class being more extensive, which may have influenced the model’s predictive accuracy. Second, the higher predictive accuracy of stable orchard meadows compared to orchard meadow losses may have been the result of heterogeneity in drivers of orchard meadow loss (as highlighted by our results) compared to the more homogeneous environmental and socio-economic characteristics associated with stable orchards.

## Conclusions

Scattered trees are keystone structures for biodiversity and ecosystem services in many agricultural landscapes of the world. We found that the total area of orchard meadows, one of the most common types of scattered trees in Europe, has decreased in our study area by one fifth over between 1968 and 2009. Our study explained the spatial pattern of losses by a set of environmental, demographic, and socio-economic driving factors in a boosted regression model. Testing five hypotheses, we found that orchard meadow loss occurred in areas of smoother slopes (H1), in areas where smaller plot sizes and fragmented orchard meadows prevailed (H2), and in areas near settlements and infrastructure (H4). The analysis did not confirm our hypotheses that orchard meadow loss would be higher in areas where agricultural intensification was stronger (H3) and in areas of lower implementation levels of conservation policies (H5). Our model confirmed that the more influential drivers of orchard meadow loss were those contributing towards their reduced economic profitability and increased opportunity costs for keeping them, providing incentives for conversion of orchard meadows to other, more profitable and simplified land uses [15,58]. In particular, this study has highlighted the role of fragmentation as a driver of orchard meadow loss.

The developments that we found—a 22% loss of orchard meadow area and a 25% loss of orchard meadow plots—are likely to have negative consequences on farmland biodiversity, affecting, for example, bird, bee and wasp, beetle, butterfly, land snail, and plant populations [59], and especially the habitat specialists within these groups [60]. Agriculture and urbanization are powerful proximate causes for these developments, but both are extremely challenging to address through conservation policies at local and regional scales. However, our study gives insights about the spatial location of the most persistent types of orchard meadows in agricultural landscapes. Orchard meadows with the least likelihood of being converted are: those on steeper slopes; those on larger parcels, with larger plot sizes, and with less fragmentation; and those in distance to settlements and roads. (i.e. those areas that are least likely to be converted). As biodiversity conservation in orchard meadows requires permanent and cost-intensive management interventions (for example, regular pruning and planting of fruit trees), these insights could be taken up by conservation policies for a spatial priorization of conservation efforts.

## Supporting Information

S1 Table Dataset underlying the findings: dependent and explanatory variables at point and municipality levels. (XLSX)

S2 Table Confusion matrix of predicted and observed values for occurred orchard meadow loss. (DOCX)

S3 Table Identified variable interactions. (DOCX)
