# Supplementary material for: Patterns and Drivers of Scattered Tree Loss in Agricultural Landscapes: Orchard Meadows in Germany (1968-2009)
Source: PLoS One. 2015 May 1;10(5):e0126178. doi: 10.1371/journal.pone.0126178 (PMC4416762; doi:10.1371/journal.pone.0126178)
Supplement: S2 Table — (DOCX) [file pone.0126178.s002.docx]

|  |  | **Predicted** | | **Sum** |  |
| --- | --- | --- | --- | --- | --- |
|  |  | 0 | 1 |  | |
| **Observed** | 0 | 1,535 | 27 | 1,562 | |
|  | 1 | 163 | 272 | 435 |  |
| **Sum** |  | 1,698 | 299 | 1,997 | |
