# Supplementary material for: Patterns and Drivers of Scattered Tree Loss in Agricultural Landscapes: Orchard Meadows in Germany (1968-2009)
Source: PLoS One. 2015 May 1;10(5):e0126178. doi: 10.1371/journal.pone.0126178 (PMC4416762; doi:10.1371/journal.pone.0126178)
Supplement: S3 Table — (DOCX) [file pone.0126178.s003.docx]

| **Variable 1** | **Variable 2** | **Interaction size** |
| --- | --- | --- |
| Change in number of full-time farms | Size of orchard meadow plot | 4.62 |
| Parcel size | Slope | 3.72 |
| Distance to urban settlements | Slope | 3.39 |
| Distance to secondary roads | Size of orchard meadow plot | 3.31 |
| Distance to urban settlements | Soil quality | 2.81 |
| Change in number of residential buildings | Population change | 2.64 |
| Perimeter-area ratio of orchard meadow plot | Distance to urban settlements | 2.63 |
| Change in number of part-time farms | Slope | 2.28 |
| Change in number of residential buildings | Change in number of full-time farms | 2.21 |
| Share of people ≥ 65 years among population | Size of orchard meadow plot | 2.15 |
| Perimeter-area ratio of orchard meadow plot | Slope | 2.06 |
| Change in number of livestock farms | Change in number of full-time farms | 2.06 |
| Change in number of full-time farms | Slope | 1.77 |
| Distance to urban settlements | Size of orchard meadow plot | 1.76 |
| Perimeter-area ratio of orchard meadow plot | Distance to secondary roads | 1.69 |
| Perimeter-area ratio of orchard meadow plot | Share of people ≥ 65 years among population | 1.63 |
